# George Ayer, M. D.

**Published:** 1878-01

**Authors:** 


### GEORGE AYER, M. D.

MEETING OF THE ERIE-COUNTY MEDICAL SOCIETY.—TRIBUTE OF RESPECT.

A meeting of the Erie-County Medical Society was held at the Medical College on Saturday evening, Dec. 8, '77, for the purpose of taking action on the death of the late Dr. George Ayer. The meeting was called to order by Dr. E. Storck, Vice-President of the Society, and Dr. D. W. Harrington acted as Secretary. The object having been stated by Dr. Storck, the meeting was addressed as follows by Dr. Julius F. Miner:

*Mr. President and Gentlemen:*

We gather this evening where we have so recently and so frequently met on similar occasions, to take action upon the death of a brother; and it may be proper that I, who knew the deceased more intimately than many of you, say a few words which will give a larger expression of the grief we feel in the loss we have sustained in the death of our respected friend and associate, Dr. George Ayer. Dr. Ayer was born in Hampton, N. H., in May, 1821, and by his own unaided efforts fitted himself for college and was graduated from Dartmouth in 1841. Three years later he graduated from the medical department of the same institution. He located in Stafford, Genesee County, N. Y. where he practised his profession for about eighteen years, and from there he removed to Buffalo in 1863, since which time I have known him intimately in a professional way, and have respected his opinions and admired his confidence and trust in the truthfulness of his conclusions. He was open to new truth, but when his opinion was formed it was not easily changed. He had in this city an extensive and quite lucrative practice, enjoying the confidence and respect of his numerous patrons and friends in a very marked degree. He was an educated, faithful and skillful physician, and will be truly mourned by a wide circle of patrons and friends.

He has labored earnestly and faithfully and fallen a victim to the dangers and hardships of the profession which he ardently loved and most truly honored. In his devotion to and care of the poor and abandoned, he contracted blood poison of great activity and virulence, in 1872, which cast the shadow of death over all the remainder of his life and finally ended his days.

It may interest you to know more definitely the course and progress of his malady. He was poisoned in obstetric attendance in June, 1872, and secondary symptoms manifested themselves very early in characteristic form. The deep ulcerations and cutaneous eruptions of tertiary stage soon blended with and aggravated the secondary symptoms. Impressed with the severity and danger of his disease, he consulted the most experienced surgeons of this country, following their advice, however, with little apparent benefit. In October last he was precipitated with his carriage down an embankment, a distance of twenty feet, receiving a severe shock and fright from which he never recovered. For the past three weeks he complained of severe pain in his head, back and limbs, with constipation, loss of appetite, vomiting, jaundice, etc., resulting in great exhaustion and finally in death.

After the conclusion of Dr. Miner's address, remarks were made by Drs. W. W. Miner, and F. W Bartlett, when a committee consisting of Drs. J. F. Miner, J. C. Green and C. C. Wyckoff, was appointed to draft a memorial expressing the sentiment of the Society upon the death of Dr. Ayer. The committee reported the following, which was adopted :

MEMORIAL :

Dr. George Ayer, at the close of a laborious, earnest, successful and honorable life, is peacefully at rest. He died December 7th, 1877, at the age of fifty-six years, cut off untimely by one of the accidents peculiar to our calling. He was conscious to the last, talking calmly and discriminately with his friends of the great change of death and the hopes of a final rest. He was an honest, upright, earnest, intelligent worker in the profession and had traits of character to be admired and imitated. Though conscious of having enjoyed superior advantages of education and observation, he was retiring and unassuming in manner. He was true and faithful to his friends, and ever willing to forgive wrongs. We may be proud that he had so long and faithfully labored among us, and endeavor to emulate his virtues and profit by his example.

On motion the Society resolved to attend the funeral of Dr. George Ayer in a body, wearing the usual badge of mourning. The meeting then adjourned.

